# The Impact of COVID-19 Lockdown on Daily Activities, Cognitions, and Stress in a Lonely and Distressed Population: Temporal Dynamic Network Analysis

**DOI:** 10.2196/32598

**Published:** 2022-03-17

**Authors:** Matthias Haucke, Andreas Heinz, Shuyan Liu, Stephan Heinzel

**Affiliations:** 1 Department of Psychiatry and Neurosciences Charité–Universitätsmedizin Berlin Campus Charité Mitte Berlin Germany; 2 Clinical Psychology and Psychotherapy Department of Education and Psychology Freie Universität Berlin Berlin Germany

**Keywords:** COVID-19, mental health, outbreak, epidemic, pandemic, psychological response, emotional well-being, ecological momentary assessment, risk, protective factors, lockdown measures, loneliness, mood inertia, stressors, mobile apps, mHealth, digital health, EMA, smartphone apps, network model, cognition, stress, temporal dynamic network, permutation testing, network comparison, network characteristics, multilevel vector autoregressive model, mlVAR

## Abstract

**Background:**

The COVID-19 pandemic and its associated lockdown measures impacted mental health worldwide. However, the temporal dynamics of causal factors that modulate mental health during lockdown are not well understood.

**Objective:**

We aimed to understand how a COVID-19 lockdown changes the temporal dynamics of loneliness and other factors affecting mental health. This is the first study that compares network characteristics between lockdown stages to prioritize mental health intervention targets.

**Methods:**

We combined ecological momentary assessments with wrist-worn motion tracking to investigate the mechanism and changes in network centrality of symptoms and behaviors before and during lockdown. A total of 258 participants who reported at least mild loneliness and distress were assessed 8 times a day for 7 consecutive days over a 213-day period from August 8, 2020, through March 9, 2021, in Germany, covering a “no-lockdown” and a “lockdown” stage. COVID-19–related worry, information-seeking, perceived restriction, and loneliness were assessed by digital visual analog scales ranging from 0 to 100. Social activity was assessed on a 7-point Likert scale, while physical activity was recorded from wrist-worn actigraphy devices.

**Results:**

We built a multilevel vector autoregressive model to estimate dynamic networks. To compare network characteristics between a no-lockdown stage and a lockdown stage, we performed permutation tests. During lockdown, loneliness had the highest impact within the network, as indicated by its centrality index (ie, an index to identify variables that have a strong influence on the other variables). Moreover, during lockdown, the centrality of loneliness significantly increased. Physical activity contributed to a decrease in loneliness amid the lockdown stage.

**Conclusions:**

The COVID-19 lockdown increased the central role of loneliness in triggering stress-related behaviors and cognition. Our study indicates that loneliness should be prioritized in mental health interventions during lockdown. Moreover, physical activity can serve as a buffer for loneliness amid social restrictions.

## Introduction

The outbreak of COVID-19 is an unprecedented global health challenge; as of November 2021, there are 259,502,031 confirmed cases and 5,183,003 deaths globally [1]. To mitigate the spread of SARS-CoV-2, most countries enforced lockdown measures, including social restrictions, travel bans, stay-at-home orders, and business shutdown. Together with the pandemic per se, these lockdown measures increased global mental health problems [2,3]. Reasons for this are an increase of distress or loneliness during the COVID-19 lockdown [4-7], yet most studies are overlooking the directionality between behavior and cognition over time. Recently, a network approach to psychopathology proposed that changes in mental health result from a temporal dynamic interaction between mental states, such that one mental state at one moment in time (eg, worry) can trigger other mental states at the next moment in time (eg, feeling stressed) [8]. We set out to examine whether lockdown measures can alter the dynamic network structure of behavior (eg, physical activity) and pandemic-related mental states (eg, worry). To do so, we compared differences between moment-to-moment time-lagged associations of pandemic-related cognitions, behaviors, and mental health, and tested for changes in centrality between lockdown stages. Comparing centrality (ie, an index to identify variables that have a strong influence on the other variables) can be informative in finding the most protective or detrimental temporal influence on mental health amid a lockdown [9,10]. This knowledge can be transferred to prioritize targets for pandemic-related mental health care interventions.

Psychological distress and social isolation are risk factors for developing mental disorders [11-15]. Therefore, we focused on a subpopulation who were experiencing at least mild levels of psychological distress and loneliness amid the COVID-19 pandemic. Moreover, we gathered real-life data using ecological momentary assessments (EMAs) via smartphone technology and measured objective physical activity via wrist-worn actigraphy devices. We investigated the temporal associations between loneliness, stress, physical and social activity, and COVID-19–related behaviors and cognitions.

We measured three COVID-19–related cognitions: perceived restriction in everyday life due to the pandemic, seeking information about the pandemic, and worrying about the pandemic’s impact on one’s life. Worries about the COVID-19–related economic downfall and the possible health impact on oneself or others can increase psychological distress [7,16]. In addition, distress, anxiety, depression, and anger are further increased by physical and social distancing measures [17,18]. People who stayed at home often acquired more COVID-19–related information through digital media, which increased anxiety and psychological distress [19-22]. Thus, COVID-19–related worrying, perceptions of restrictions, and information-seeking can be central causes of mental health issues.

Prior to the COVID-19 pandemic, loneliness was already recognized as one of the most pressing issues in modern societies [23]. Loneliness is an aversive state resulting from a discrepancy between an individual’s desired and realized social relationships [24]. Limiting social contacts and closing off social spaces can help to halt the spread of COVID-19; however, they also increase feelings of loneliness [7,25]. Loneliness has serious consequences for health, including increasing the risk of cardiovascular disease and immune dysfunction, depression, anxiety, and suicidal ideation [26]. To buffer against feelings of loneliness during lockdown, it can be essential to receive social support and engage in digital social activities [27,28].

A second buffer against mental health problems during the pandemic might be physical activity. Physical activity can relieve stress [29]; enhance cognitive abilities [30]; and reduce the risk of diabetes [31], cardiovascular disease [32], cancer [33], and mental disorders [34,35]. Conversely, sedentary behavior, defined as low-energy-expenditure behavior (≤1.5 metabolic equivalents), increases the risk for negative health outcomes, including type 2 diabetes mellitus, cardiovascular disease, and all-cause mortality [36-38]. Physical activity can lead to physiological reactions associated with decreased depression, such as an increase in neuroplasticity, cerebral blood flow, delivery of neurotrophic factors and oxygen, and resistance to oxidative stress [39]. Finally, exercise can improve self-efficacy and self-esteem [40]. We assessed physical activity through actigraphy (ie, a wrist-worn device that obtains objective measures of physical activity) [41].

Our study was performed in Germany during a no-lockdown stage (August 8 to November 1, 2020) and a lockdown stage (November 2, 2020, to March 9, 2021). During the no-lockdown stage, the restrictions were lenient (eg, no private or public meeting restrictions, and leisure facilities, bars, and catering facilities were open). To counter the steep increase in active COVID-19 cases, the German government announced a lockdown on November 2, 2020, including social restrictions, travel bans, closing of restaurants and cinemas, and business shutdowns. In addition, these lockdowns measures were further tightened on December 16 (eg, closing of most retail; see Supplement A in Multimedia Appendix 1).

The aim of this study was to examine the temporal dynamic interplay between COVID-19 pandemic–related cognitions, behaviors, and mental health states. This is the first study to use a dynamic network approach to compare moment-to-moment time-lagged associations between pandemic-related cognitions, behaviors, and mental health states between lockdown stages. Moreover, we examined whether the lockdown affects the centrality of loneliness and specific pandemic-related behaviors and cognitions (ie, a more central variable has more and stronger connections to other variables). This helps to identify the most protective or detrimental influences on mental health during a lockdown. This knowledge, in turn, can be used to prioritize mental health intervention targets. Specifically, we hypothesized that a lockdown, in comparison to a no-lockdown period, increases the centrality of stress, physical activity, social contacts, and loneliness. Finally, we hypothesized that stress and loneliness will have a stronger influence on COVID-19–related behaviors and cognitions during lockdown than during no-lockdown.

## Methods

### Participants and Sampling

**Figure 1:**
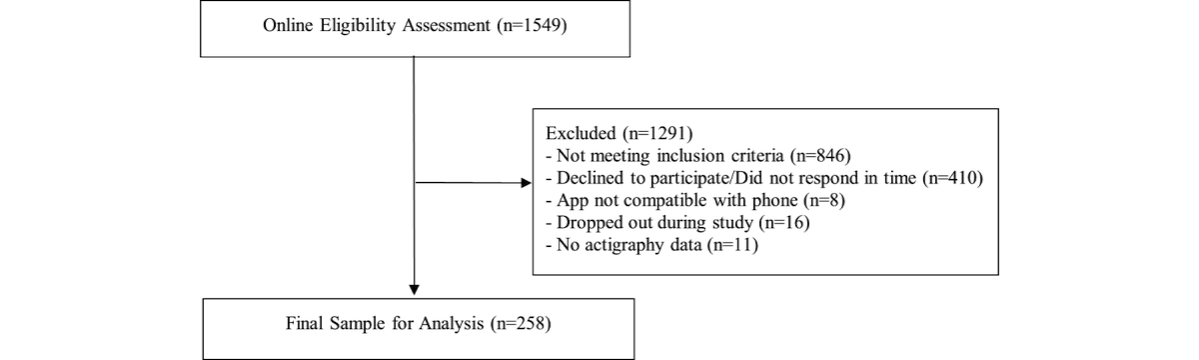
Recruitment flow.

We assessed 1549 participants for eligibility in an online questionnaire. The final sample size was 258 (see Figure 1 for the recruitment flow). On average, participants missed 17.5% of the questionnaires, no participants missed more than 50% of the sent questionnaires, and 117 data points were marked by the GGIR package [42] as “nonwear” and subsequently excluded from the analyses. Specifically, the accelerometer nonwear score was estimated based on the standard deviation and range of the raw data from each accelerometer axis [42].

Inclusion criteria were (1) a minimum age of 18 years, (2) not working a night shift, (3) not being infected by COVID-19, (4) using an Android smartphone, and (5) speaking fluent German. Moreover, we targeted individuals who reported (6) perceived mild to moderate psychological distress and (7) sometimes felt lonely during the COVID-19 pandemic. We used the COVID-19 Peritraumatic Distress Index (CPDI [43]; cut-off score=28, indicating mild distress) questionnaire and the short-form of the University of California Los Angeles Loneliness Scale (ULS-8 [44]; cut-off score=16, indicating mild loneliness), respectively. The CPDI was designed for evaluating changes in mental health status, cognitive skills, avoidance and compulsive behavior, physical symptoms, and loss of social functioning due to the COVID-19 pandemic. The questionnaire has been previously validated in Germany [43].

### Study Design and Procedure

The study was conducted in Germany over a 213-day period between August 8, 2020, and March 9, 2021, covering a no-lockdown and a lockdown stage. Participants were recruited via online advertisements on university websites, Twitter, and eBay classifieds. Participants had to fill in an online screening questionnaire on the Siuvo Intelligent Psychological Assessment Platform. After an initial contact via phone or email, we sent participants our study information, accelerometer, informed consent, and a QR code (to install a smartphone app) by mail. After they completed the study, participants sent back the study material by mail.

We conducted the EMA via the smartphone app “movisensXS” (movisens GmbH, Karlsruhe, Germany) developed for research purposes. This app is compliant with the General Data Protection Regulation (European Union) and Berlin Data Protection Act (Berliner Datenschutzgesetz). The app consists of a sociodemographic assessment (eg, age, gender, and years of education) and measures participants’ current experiences in real time. Participants filled in questionnaires for 7 consecutive days, in which they received 8 prompts (randomized within 1 hour and 45-minute blocks between 8 AM and 10 PM). We performed an EMA that involves repeated sampling of individuals’ current behaviors and experiences in real time and in their natural environments. EMA minimizes recall bias, maximizes ecological validity, and allows approximating temporal causality (ie, Granger causality) [45]. A time series X is said to Granger-cause Y if it can be shown, usually through a series of *t* tests and *F* tests on lagged values of X (and with lagged values of Y also included), that the X values provide statistically significant information about future values of Y [46].

Moreover, we measured physical activity via the “GENEActiv” Original (Activinsights) monitor (dynamic range ±8 g, sampling frequency range 10-100 Hz). Participants wore the actigraphy device on the left wrist.

### Ethical Considerations

The study was approved by the ethics committee at Charité–Universitätsmedizin Berlin (reference EA2/143/20) and Freie Universität Berlin (reference 030/2020).

### Measures

#### EMA Items

Stress was measured with the following question: “In this moment I feel stressed.” Other items started with “During the last hour...” followed by “to which extent did you feel constrained by the pandemic in your everyday life?” (perceived restriction), “to which extent did you worry about how the pandemic affects your personal situation?” (worry), “to which extent did you seek information about the Corona pandemic?” (information-seeking), and “to which extent did you feel lonely” (loneliness). Each of these items was measured on a visual analog scale (0-100: 0=not at all, 100=most frequent or severe). Duration of social activity was measured with the question “How long did your last social contact last?” via a Likert scale ranging from 1=“0 minutes” to 7=“50-60 minutes.”

#### Actigraphy Data

Physical activity data were collected using the actigraphy devices worn by each participant on the left wrist.

### Statistical Analysis

#### Overview

All analyses were performed using R statistical software (version 3.5.3). In this section, we describe the data preparation procedures, averaged values of our measured items, estimation of the dynamic networks, and the permutation procedure used to test for group differences in centrality indices and dynamic association.

#### Data Preparation

We calculated the Euclidean norm (vector magnitude) of the raw signals of the three-measurement axis, which is a summary score of body acceleration and a validated measure for physical activity [47]. The Euclidean norm minus one (ENMO) is defined as *r_i_*–1000 [48], where




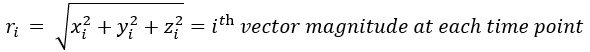




The actigraphy data from GENEActiv (100 Hz; .bin files) were downloaded using GENEActiv PC software V3.3. The GENEActiv .bin files were then exported into R statistical software V4.0.3 for processing using the GGIR package V1.2-0. We autocalibrated the raw triaxial accelerometer signals and computed the average ENMO metric for 1 hour before each beep. To exclude time frames in which participants did not wear their actigraphy device, we used the nonwear score of the GGIR package. We excluded time frames above the cut-off score of 1. As the EMA items were nonnormally distributed, we transformed all variables using the nonparanormal transformation [49]. To test for nonstationarity, we calculated a two-level autoregressive model for each lockdown group, in which each score of the variable included in our model was regressed on the immediately preceding score of that variable (ie, moment-to-moment inertia). A moment-to-moment inertia value larger than 1 indicates a nonstationary process [50]. We assumed stationarity, as the average moment-to-moment inertia ranged between 0.13 and 0.37 for all 7 included variables for each lockdown group (see Supplement B in Multimedia Appendix 1). In addition, a Kwiatkowski-Phillips-Schmidt-Shin (KPSS) test was performed separately for every subject and variable. The KPSS test indicated that the data were stationary (approximately 99.9%). The R code of the statistical analyses is available online [51].

#### Dynamic Network Estimation

We built a first-order vector autoregressive model (VAR) with the R package mlVAR. Each variable at time point *t* was predicted by all variables (including itself) at the next time point of measurement (lag 1). The results of the network models consisted of nodes (variables) and directed edges (statistical relations) that were visualized via the R package qgraph [52]

#### Permutation Testing of Centrality Indices and Edge Differences

Permutation tests were used to compare individual path and network centrality between the lockdown and no-lockdown stages. The permutation procedure was developed by Wolfgang Viechtbauer and compares the results of the observed data with a distribution derived from repeated permutation (100,000) of the data under the null hypothesis [53-55]. To assess the importance of specific variables in the network of two groups, in-strength and out-strength were calculated from all (including nonsignificant) edges in the network. In-strength reflects the sum of ingoing edge weights, whereas out-strength reflects the sum of outgoing edge weights to the specific node [56,57]. A detailed description of the permutation procedures can be found in Supplement C in Multimedia Appendix 1.

## Results

### Sociodemographics

Sociodemographic characteristics of the final sample (N=258), as well as results of independent *t* tests or *χ^2^* tests comparing these characteristics between a no-lockdown and lockdown stage are shown in Table 1. As we had more women in our lockdown group, we tested the effect of gender on all measured variables. We found that, except for social duration, gender did not significantly affect our variables (see Supplement G in Multimedia Appendix 1).

**Table 1:** Sociodemographic characteristics of participants.

Characteristic			Total (August 8, 2020, to March 9, 2021; N=258)		No-lockdown period (August 8 to November 1, 2020; n=131)		Lockdown period (November 2 to March 9, 2021; n=127)		*P* value^a^	
Age (years), mean (SD)			30.78 (11.16)		31.18 (10.52)		30.16 (11.67)		.55	
Education (years), mean (SD)			15.28 (3.69)		15.1 (3.69)		15.46 (3.69)		.44	
**Gender, n (%)**										.008
	Male	77 (29.8)		49 (37.4)		28 (22.0)				
	Female	178 (70.0)		82 (62.6)		96 (75.6)				
	Diverse	3 (1.2)		0 (0)		3 (2.4)				
**Family status, n (%)**										.93
	Single	114 (44.2)		61 (46.6)		53 (41.7)				
	In relationship	92 (35.7)		45 (34.4)		47 (37.0)				
	Married	48 (18.6)		23 (17.6)		25 (19.7)				
	Other	4 (1.6)		2 (1.5)		2 (1.6)				
Number of children, mean (SD)			1.77 (0.78)		1.7 (0.78)		1.88 (0.78)		.38	
Number living with others, mean (SD)			2.56 (2.15)		2.5 (1.29)		2.62 (2.77)		.65	
Health status (1=very bad, 5=very good), mean (SD)			3.74 (0.86)		3.65 (0.91)		3.83 (0.81)		.09	
COVID-19 risk group, n (%)			64 (24.8)		33 (25.2)		31 (24.4)		.80	
COVID-19 distress (CPDI^b^), mean (SD)			47.56 (14.79)		48.32 (16.34)		46.76 (13.31)		.41	
Loneliness (ULS-8^c^), mean (SD)			22.57 (3.97)		22.01 (4.01)		23.15 (3.85)		.02	

^a^Based on independent *t* test or *χ^2^* test; unequal variance was assumed, and we applied the Welsh approximation to the degrees of freedom.

^b^CPDI: COVID-19 Peritraumatic Distress Index.

^c^ULS-8: University of California Los Angeles Loneliness Scale.

### Average-Based Lockdown Differences

To compare the no-lockdown and lockdown stages, we performed independent *t* tests using overall averages for each person. As shown in Table 2, the lockdown significantly increased COVID-19 worries, perceived restriction, and duration of social contacts. Moreover, the lockdown significantly decreased physical activity. There was no statistically significant influence of lockdown on information-seeking, stress, and loneliness.

**Table 2:** Differences between no-lockdown and lockdown stages.

Variables		No-lockdown period (n=131), mean (SD)	Lockdown period (n=127), mean (SD)	*P* value^a^
**EMA**b** items**				
	Loneliness	22.62 (20.82)	21.45 (19.80)	.64
	COVID-19 worries	24.59 (18.36)	29.12 (17.33)	.04
	COVID-19 perceived restriction	23.86 (17.83)	28.16 (17.05)	.05
	COVID-19 information-seeking	22.85 (15.57)	23.46 (13.94)	.74
	Social contacts	2.64 (0.95)	3.05 (1.00)	<.001
	Stress	35.05 (18.43)	33.25 (17.34)	.42
Physical activity from actigraphy (microgravity)		40.15 (13.37)	35.24 (11.42)	.002

^a^*t* test; unequal variance was assumed and we applied the Welsh approximation to the degrees of freedom.

^b^EMA: ecological momentary assessment.

### Network Estimation

We wanted to investigate how a lockdown affects the temporal dynamics of pandemic-related cognitions, behaviors, and mental health states. To do so, we first estimated the temporal (ie, time-lagged) and bidirectional associations between detrimental and beneficial factors via multilevel VAR models [58-60]. These VAR models were then used to estimate temporal dynamic networks for a lockdown and a no-lockdown stage. Permutation testing was used to test for group differences in individual paths and the network centrality of pandemic-related detrimental and beneficial mental health factors between the lockdown and no-lockdown stage. Moreover, the exploratory results of a permutation test for the difference in overall connectivity are provided in Supplement C of Multimedia Appendix 1.

### Edge Differences Between Groups

**Figure 2:**
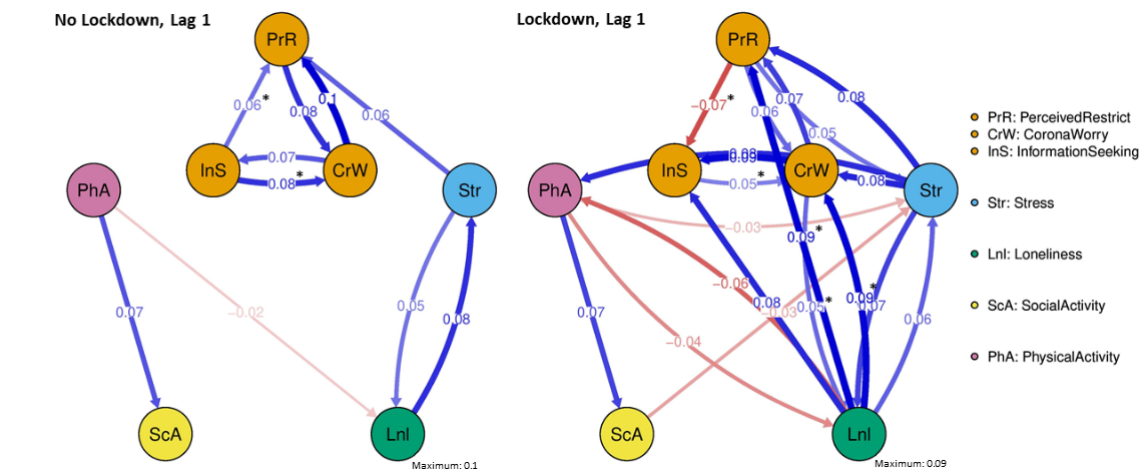
Temporal dynamic networks for a no-lockdown and a lockdown stage. Temporal relations among ecological momentary assessment and physical activity data, measured by actigraphy devices, estimated with a multilevel vector autoregressive model, and depicted as a graph where nodes are variables and edges (arrows connecting nodes) are statistically significant (α<.05) partial correlations among variables. Thicker and more saturated edges depict stronger relations; positive relations are in blue and negative relations are in red. Associations that are significantly different between the no-lockdown and lockdown stages (permutation testing using a two-sided *P* value at the uncorrected α level) are marked with an asterisk.

Figure 2 displays the “full” dynamic symptom networks for the lockdown and no-lockdown groups, which include only statistically significant *edges* (ie, time-lagged partial correlations with α<.05). Permutation tests revealed that 7 of the edges were significantly different between the no-lockdown and lockdown groups at the uncorrected α level (indicated with an asterisk in Figure 2).

Compared to no-lockdown, in a lockdown, participants showed a stronger connection from “loneliness” to “perceived restriction” (difference –0.114, *P*<.001) and from “loneliness” to “COVID-19–related worry” (difference –0.0767, *P*=.03).

Compared to no-lockdown, in a lockdown, participants showed a weaker connection from “information-seeking” to “perceived restriction” (difference 0.0609, *P*=.02) and from “information-seeking” to “COVID-19–related worry” (difference 0.0477, *P*=.05). In addition, information-seeking led to less information-seeking in the next moment (ie, weaker autocorrelation; difference 0.0754, *P*=.02).

Compared to no-lockdown, during the lockdown, participants showed a stronger connection from “COVID-19–related worry” to “loneliness” (difference –0.0444, *P*=.05).

Compared to no-lockdown, during the lockdown, participants showed a weaker connection from “perceived restriction” to “social activity” (difference 0.0065, *P*=.01).

More information on the time-lagged partial correlations (ie, edges) that were significantly different during the lockdown can be found in Table 3 (all, including nonsignificant, edge differences are shown in Supplement F of Multimedia Appendix 1).

**Table 3:** Significant edge differences of time-lagged partial correlation coefficients between the lockdown and no-lockdown stages.

Predictor (1–lag)	Outcome	Partial correlation coefficient		Difference in partial correlation coefficient	*P* value
		No-Lockdown	Lockdown		
Information-seeking	Perceived restriction	0.0548	–0.0062	0.0609	.02
Loneliness	Perceived restriction	0.001	0.115	–0.114	<.001
Information-seeking	COVID-19–related worry	0.0689	0.0212	0.0477	.05
Loneliness	COVID-19–related worry	0.0274	0.1042	–0.0767	.03
Information-seeking	Information-seeking	0.1721	0.0967	0.0754	.02
COVID-19–related worry	Loneliness	–0.0129	0.0315	–0.0444	.05
Perceived restriction	Social activity	0.0043	–0.0021	0.0065	.01

### Centrality Indices

*In-strength* is the sum of *ingoing* edge weights to a specific node and *out-strength* is the sum of the *outgoing* edge weights to a specific node. During the no-lockdown stage, worrying about COVID-19 had the highest out-strength, indicating that when a participant reports worries about COVID-19 at one measurement occasion, it is likely that this participant will report other COVID-19–related behaviors and cognitions at the next measurement occasion. During lockdown, loneliness had the highest out-strength, indicating that when a participant reports feeling lonely in one moment, this participant is likely to report COVID-19–related behaviors and cognitions in the next momentary assessment.

**Figure 3:**
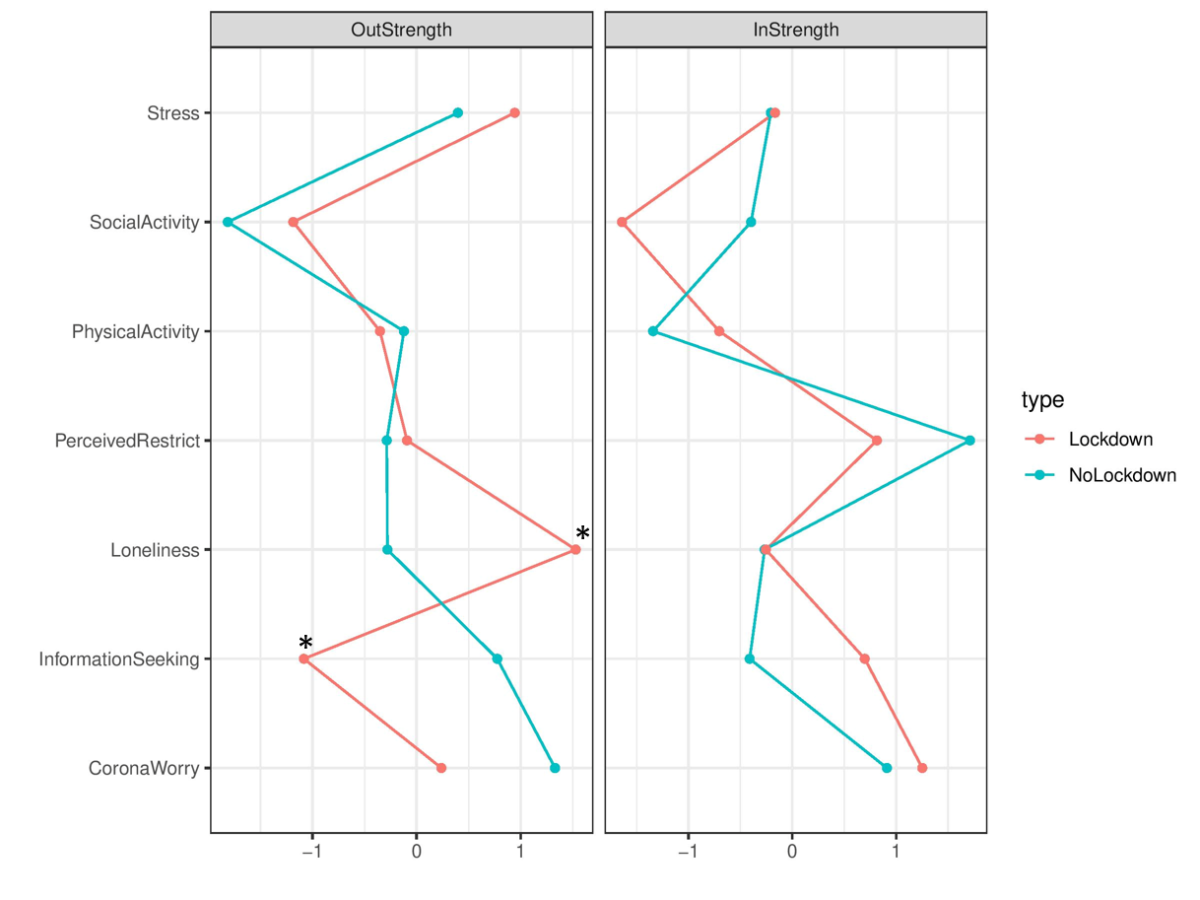
The standardized centrality indices out-strength and in-strength among ecological momentary assessment and physical activity data within the networks of the no-lockdown and lockdown stages. The statistically significant indices (permutation tests using a two-sided *P* value at the uncorrected α level) are marked with asterisks.

Permutation tests revealed a significant higher out-strength for “loneliness” during lockdown (difference –0.1975, *P*=.04) and significant lower out-strength for “information-seeking” (difference 0.1452, *P*=.03) at the uncorrected α level (as indicated by asterisks in Figure 3). More information on centrality indices that were significantly different can be found in Table 4 (all, including nonsignificant, differences between centrality indices can be found in Supplement E of Multimedia Appendix 1).

**Table 4:** Significant differences in variable out-strength between lockdown and no-lockdown stages.

Variable	Out-strength			Difference		*P* value
	No-lockdown	Lockdown				
Information-seeking	0.3129	0.1677	0.1452		.03	
Loneliness	0.4109	0.6084	–0.1975		.04	

## Discussion

### Principal Findings

The COVID-19 pandemic increased mental health problems worldwide [2,61]. Our study sheds light on the mechanisms with which a lockdown affects mental health during the COVID-19 pandemic. Compared to no-lockdown, during lockdown, loneliness had a stronger impact on pandemic-related cognitions and behaviors such as perceived restrictions and worries about the pandemic. In turn, pandemic-related cognitions and behaviors reinforced each other and increased stress across lockdown stages. Finally, we found engaging in daily physical activity to be an effective strategy against feelings of loneliness during lockdown. In sum, our results suggest that when strict lockdown measures are in place, loneliness is the central trigger of stress-related behaviors and cognitions. Thus, loneliness should be prioritized in mental health interventions in the context of pandemic-related psychological distress.

Loneliness is a distressing emotional state in which one experiences a discrepancy between the desired and perceived quantity and quality of social relations [62]. Previous studies showed that lonely individuals exhibit a negative information bias such as increased attention for social threatening stimuli, negative and hostile intent attributions, expectation of rejection, and rumination [63]. We found that during lockdown, feelings of loneliness had the highest out-strength, indicating that loneliness is the central trigger of stress-related behaviors and cognitions. Compared to a no-lockdown, a lockdown increased the out-strength of loneliness, which indicates that loneliness has a more central role in affecting stress-related cognitions and behaviors during lockdown. Moreover, during lockdown, the influence of loneliness on perceptions of restriction and COVID-19–related worry increased. Thus, a lockdown changes the way loneliness interacts with pandemic-related behaviors and cognitions.

COVID-19–related-worries, feelings of restriction, and information-seeking were mutually reinforcing over time in both the no-lockdown and lockdown stages, resulting in a vicious stress-inducing cycle from which it can be increasingly difficult to escape. Information-seeking had less out-strength during lockdown compared to the no-lockdown stage, which indicates that COVID-19–related information-seeking has a more central role during a no-lockdown period. During lockdown, information-seeking at one moment led to less information-seeking at the next moment (ie, weaker autocorrelation), and its influence on perceived restrictions and COVID-19–related worry decreased. These findings contrast earlier reports concluding a more significant influence of information-seeking during lockdown, based on findings of increased averaged information-seeking [19,21]. Moreover, during the no-lockdown stage, “feeling restricted” increased information-seeking, whereas during lockdown, “feeling restricted” decreased information-seeking. This suggests that during a no-lockdown stage, people are in a type of information-approach state, whereas during lockdown, people are more likely to be in an information-avoidance state. Therefore, the best moment to communicate COVID-19–relevant information such as safety behaviors might be an early pandemic stage when no lockdown measures are in place.

Physical activity increased social activity in both the no-lockdown and lockdown stages. This association might result from public health recommendations that suggest meeting people only outside enclosed spaces. During COVID-19, people might have combined physical and social activity (ie, they found a companion to go for a walk or hike outside). Physical activity can also help to form interpersonal relationships (eg, attending a virtual group fitness class). Moreover, physical activity decreased feelings of loneliness during lockdown. A possible reason is that physical activity can mediate contextual influences on loneliness (eg, being in nature and physically active rather than sitting at home and leading a sedentary lifestyle) [64]. Meeting more people did not decrease feelings of loneliness in either of the lockdown stages. A potential explanation is that feelings of loneliness are not caused by the number of social contacts but rather the perception that current relationships do not match desired relationships (eg, the other person being attentive to one’s needs) [65]. Finally, physical activity and social activity were associated with decreased stress only during the lockdown stage, indicating that during lockdown, these stress-buffering behaviors become effective.

### Perspectives on Mental Health Interventions

We found that loneliness has the highest temporal effect on all measured moment-to-moment pandemic-related cognition and behaviors during lockdown. This, in turn, suggests that loneliness can be a central trigger of stress-related behaviors and cognitions. Our study suggests that mental health interventions during the pandemic lockdown should prioritize the feeling of loneliness rather than pandemic-related rumination, feelings of restriction, or information-seeking. This could be achieved by a digital mental health approach (eg, online therapy or smartphone-based interventions) that fosters a sense of belonging and community [66-70]. To our knowledge, this is the first study to use a temporal network model comparison approach to identify and refine mental health intervention targets. This approach might be valuable to identify possible temporal causal trigger variables for negative cognitions and behaviors in other types of mental health interventions as well.

### Limitations

This was a natural experiment with high ecological validity but low control for extraneous variables, including seasonal effects [71]. Moreover, we cannot exclude the possibility that the observed interactions are influenced by other unmeasured underlying factors [72]. In addition, we have independent samples for comparisons of lockdown and no-lockdown stages. Thus, we cannot exclude the possibility that differences in sample characteristics may have influenced the results. However, except for the loneliness score and gender distribution, the samples did not differ in any of the measured variables. We assume that the slightly higher loneliness measure (ULS-8) in the lockdown sample was due to the lockdown. However, it cannot be ruled out that we recruited participants who were generally lonelier in the lockdown sample by chance. Gender did not have an influence on any of the measured variables, except for time spent on social activities. Here, women reported higher values than men or diverse genders. Taken together, it is unlikely that there is a major bias in our central findings due to differences in sample characteristics.

### Conclusion

To develop effective pandemic mental health interventions, it is crucial to understand the temporal dynamics of mental health factors during a COVID-19 lockdown. In comparison to a no-lockdown stage, a lockdown increased the central role of loneliness in triggering pandemic-related behaviors and cognition. In turn, pandemic-related cognitions and behaviors such as perceived restrictions and worries about the pandemic reinforced each other and increased stress. In addition, we found that physical activity can be an effective buffer against stress and loneliness during lockdown. Our results suggest that loneliness can be the central trigger for stress-related behaviors and cognitions during lockdown and therefore should be prioritized in mental health interventions.

## Appendix

Multimedia Appendix 1 Supplemental background, analysis methods, and data (Supplement A-G).

## Abbreviations

CPDI: COVID-19 Peritraumatic Distress Index
EMA: ecological momentary assessment
ENMO: Euclidean norm minus one
KPSS: Kwiatkowski-Phillips-Schmidt-Shin
ULS-8: University of California Los Angeles Loneliness Scale
VAR: vector autoregressive model

## References

[1] WHO coronavirus (COVID-19) dashboard. World Health Organization.

[2] Cénat JM, Blais-Rochette C, Kokou-Kpolou CK, Noorishad P, Mukunzi JN, McIntee S, Dalexis RD, Goulet M, Labelle PR (2021). Prevalence of symptoms of depression, anxiety, insomnia, posttraumatic stress disorder, and psychological distress among populations affected by the COVID-19 pandemic: A systematic review and meta-analysis. Psychiatry Res.

[3] Vindegaard N, Benros ME (2020). COVID-19 pandemic and mental health consequences: Systematic review of the current evidence. Brain Behav Immun.

[4] O'Connor RC, Wetherall K, Cleare S, McClelland H, Melson AJ, Niedzwiedz CL, O'Carroll RE, O'Connor DB, Platt S, Scowcroft E, Watson B, Zortea T, Ferguson E, Robb KA (2021). Br J Psychiatry.

[5] Ozamiz-Etxebarria N, Idoiaga Mondragon N, Dosil Santamaría M, Picaza Gorrotxategi M (2020). Psychological symptoms during the two stages of lockdown in response to the COVID-19 outbreak: an investigation in a sample of citizens in northern Spain. Front Psychol.

[6] Nelson BW, Pettitt A, Flannery JE, Allen NB (2020). Rapid assessment of psychological and epidemiological correlates of COVID-19 concern, financial strain, and health-related behavior change in a large online sample. PLoS One.

[7] Liu S, Heinzel S, Haucke MN, Heinz A (2021). Increased psychological distress, loneliness, and unemployment in the spread of COVID-19 over 6 months in Germany. Medicina.

[8] Wichers M, Wigman JTW, Myin-Germeys I (2015). Micro-level affect dynamics in psychopathology viewed from complex dynamical system theory. Emotion Review.

[9] Bringmann LF, Elmer T, Epskamp S, Krause RW, Schoch D, Wichers M, Wigman JTW, Snippe E (2019). What do centrality measures measure in psychological networks?. J Abnorm Psychol.

[10] van Gils A, Burton C, Bos EH, Janssens KA, Schoevers RA, Rosmalen JG (2014). Individual variation in temporal relationships between stress and functional somatic symptoms. J Psychosom Res.

[11] Deng W, Cheung S, Tsao S, Wang X, Tiwari A (2016). Telomerase activity and its association with psychological stress, mental disorders, lifestyle factors and interventions: A systematic review. Psychoneuroendocrinology.

[12] Heinz A, Deserno L, Reininghaus U (2013). Urbanicity, social adversity and psychosis. World Psychiatry.

[13] Narita Z, Stickley A, DeVylder J (2020). Loneliness and psychotic experiences in a general population sample. Schizophr Res.

[14] Beutel ME, Klein EM, Brähler E, Reiner I, Jünger C, Michal M, Wiltink J, Wild PS, Münzel T, Lackner KJ, Tibubos AN (2017). Loneliness in the general population: prevalence, determinants and relations to mental health. BMC Psychiatry.

[15] Heinz AJ, Beck A, Meyer-Lindenberg A, Sterzer P, Heinz A (2011). Cognitive and neurobiological mechanisms of alcohol-related aggression. Nat Rev Neurosci.

[16] Rauschenberg C, Schick A, Goetzl C, Roehr S, Riedel-Heller SG, Koppe G, Durstewitz D, Krumm S, Reininghaus U (2021). Social isolation, mental health, and use of digital interventions in youth during the COVID-19 pandemic: A nationally representative survey. Eur Psychiatry.

[17] Cosco TD, Fortuna K, Wister A, Riadi I, Wagner K, Sixsmith A (2021). COVID-19, social isolation, and mental health among older adults: a digital catch-22. J Med Internet Res.

[18] Zhang W, Yang X, Zhao J, Yang F, Jia Y, Cui C, Yang X (2020). Depression and psychological-behavioral responses among the general public in China during the early stages of the COVID-19 pandemic: survey study. J Med Internet Res.

[19] Terry PC, Parsons-Smith RL, Terry VR (2020). Mood responses associated with COVID-19 restrictions. Front Psychol.

[20] Kleiman EM, Yeager AL, Grove JL, Kellerman JK, Kim JS (2020). Real-time mental health impact of the COVID-19 pandemic on college students: ecological momentary assessment study. JMIR Ment Health.

[21] Huckins JF, daSilva AW, Wang W, Hedlund E, Rogers C, Nepal SK, Wu J, Obuchi M, Murphy EI, Meyer ML, Wagner DD, Holtzheimer PE, Campbell AT (2020). Mental health and behavior of college students during the early phases of the COVID-19 pandemic: longitudinal smartphone and ecological momentary assessment study. J Med Internet Res.

[22] Arend A, Blechert J, Pannicke B, Reichenberger J (2020). Increased screen use on days with increased perceived COVID-19-related confinements-a day level ecological momentary assessment study. Front Public Health.

[23] Jeste DV, Lee EE, Cacioppo S (2020). Battling the modern behavioral epidemic of loneliness: suggestions for research and interventions. JAMA Psychiatry.

[24] Cacioppo J, Cacioppo S (2018). Loneliness in the modern age: An evolutionary theory of loneliness (ETL). Adv Exp Psychol.

[25] Haucke M, Liu S, Heinzel S (2021). The persistence of the impact of COVID-19-related distress, mood inertia, and loneliness on mental health during a postlockdown period in Germany: an ecological momentary assessment study. JMIR Ment Health.

[26] Singer C (2018). Health effects of social isolation and loneliness. J Aging Life Care.

[27] Brooks SK, Webster RK, Smith LE, Woodland L, Wessely S, Greenberg N, Rubin GJ (2020). The psychological impact of quarantine and how to reduce it: rapid review of the evidence. Lancet.

[28] Heinz A, Zhao X, Liu S (2020). Implications of the association of social exclusion with mental health. JAMA Psychiatry.

[29] Burg MM, Schwartz JE, Kronish IM, Diaz KM, Alcantara C, Duer-Hefele J, Davidson KW (2017). Does stress result in you exercising less? Or does exercising result in you being less stressed? Or is it both? Testing the bi-directional stress-exercise association at the group and person (N of 1) level. Ann Behav Med.

[30] Hillman CH, Erickson KI, Kramer AF (2008). Be smart, exercise your heart: exercise effects on brain and cognition. Nat Rev Neurosci.

[31] Hamilton M, Hamilton D, Zderic T (2014). Sedentary behavior as a mediator of type 2 diabetes. Med Sport Sci.

[32] Myers J, McAuley P, Lavie CJ, Despres J, Arena R, Kokkinos P (2015). Physical activity and cardiorespiratory fitness as major markers of cardiovascular risk: their independent and interwoven importance to health status. Prog Cardiovasc Dis.

[33] Moore SC, Lee I, Weiderpass E, Campbell PT, Sampson JN, Kitahara CM, Keadle SK, Arem H, Berrington de Gonzalez A, Hartge P, Adami H, Blair CK, Borch KB, Boyd E, Check DP, Fournier A, Freedman ND, Gunter M, Johannson M, Khaw K, Linet MS, Orsini N, Park Y, Riboli E, Robien K, Schairer C, Sesso H, Spriggs M, Van Dusen R, Wolk A, Matthews CE, Patel AV (2016). Association of leisure-time physical activity with risk of 26 types of cancer in 1.44 million adults. JAMA Intern Med.

[34] Vancampfort D, Firth J, Schuch FB, Rosenbaum S, Mugisha J, Hallgren M, Probst M, Ward PB, Gaughran F, De Hert M, Carvalho AF, Stubbs B (2017). Sedentary behavior and physical activity levels in people with schizophrenia, bipolar disorder and major depressive disorder: a global systematic review and meta-analysis. World Psychiatry.

[35] Fox KR (2007). The influence of physical activity on mental well-being. Public Health Nutr.

[36] Biswas A, Oh PI, Faulkner GE, Bajaj RR, Silver MA, Mitchell MS, Alter DA (2015). Sedentary time and its association with risk for disease incidence, mortality, and hospitalization in adults: a systematic review and meta-analysis. Ann Intern Med.

[37] Chau JY, Grunseit AC, Chey T, Stamatakis E, Brown WJ, Matthews CE, Bauman AE, van der Ploeg HP (2013). Daily sitting time and all-cause mortality: a meta-analysis. PLoS One.

[38] Wilmot EG, Edwardson CL, Achana FA, Davies MJ, Gorely T, Gray LJ, Khunti K, Yates T, Biddle SJH (2012). Sedentary time in adults and the association with diabetes, cardiovascular disease and death: systematic review and meta-analysis. Diabetologia.

[39] Kandola A, Ashdown-Franks G, Hendrikse J, Sabiston CM, Stubbs B (2019). Physical activity and depression: towards understanding the antidepressant mechanisms of physical activity. Neurosci Biobehav Rev.

[40] Sani SHZ, Fathirezaie Z, Brand S, Pühse U, Holsboer-Trachsler E, Gerber M, Talepasand S (2016). Physical activity and self-esteem: testing direct and indirect relationships associated with psychological and physical mechanisms. Neuropsychiatr Dis Treat.

[41] Rowlands A, Fraysse F, Catt M (2015). Comparability of measured acceleration from accelerometry-based activity monitors. Med Sci Sports Exerc Jan.

[42] van Hees VT, Renström F, Wright A, Gradmark A, Catt M, Chen KY, Löf M, Bluck L, Pomeroy J, Wareham NJ, Ekelund U, Brage S, Franks PW (2011). Estimation of daily energy expenditure in pregnant and non-pregnant women using a wrist-worn tri-axial accelerometer. PLoS One.

[43] Liu S, Heinz A (2020). Cross-cultural validity of psychological distress measurement during the coronavirus pandemic. Pharmacopsychiatry.

[44] Hays R, DiMatteo MR (1987). A short-form measure of loneliness. J Pers Assess.

[45] Shiffman S, Stone AA, Hufford MR (2008). Ecological momentary assessment. Annu Rev Clin Psychol.

[46] Hamilton JD (1994). Time series analysis.

[47] van Hees VT, Gorzelniak L, Dean León EC, Eder M, Pias M, Taherian S, Ekelund U, Renström F, Franks PW, Horsch A, Brage S (2013). Separating movement and gravity components in an acceleration signal and implications for the assessment of human daily physical activity. PLoS One.

[48] van Hees VT, Fang Z, Langford J, Assah F, Mohammad A, da Silva ICM, Trenell MI, White T, Wareham NJ, Brage S (2014). Autocalibration of accelerometer data for free-living physical activity assessment using local gravity and temperature: an evaluation on four continents. J Appl Physiol.

[49] Liu H, Han F, Yuan M, Lafferty J, Wasserman L (2012). High-dimensional semiparametric Gaussian copula graphical models. Ann Statist.

[50] de Haan-Rietdijk S, Kuppens P, Hamaker EL (2016). What's in a day? A guide to decomposing the variance in intensive longitudinal data. Front Psychol.

[51] R code for the statistical analysis. OSF.

[52] Epskamp S, Cramer AOJ, Waldorp LJ, Schmittmann VD, Borsboom D (2012). qgraph: network visualizations of relationships in psychometric data. J Stat Soft.

[53] Snippe E, Viechtbauer W, Geschwind N, Klippel A, de Jonge P, Wichers M (2017). The impact of treatments for depression on the dynamic network structure of mental states: two randomized controlled trials. Sci Rep.

[54] Klippel A, Viechtbauer W, Reininghaus U, Wigman J, van Borkulo C, Myin-Germeys I, Wichers M, MERGE (2018). The cascade of stress: a network approach to explore differential dynamics in populations varying in risk for psychosis. Schizophr Bull.

[55] Groen RN, Snippe E, Bringmann LF, Simons CJ, Hartmann JA, Bos EH, Wichers M (2019). Capturing the risk of persisting depressive symptoms: a dynamic network investigation of patients' daily symptom experiences. Psychiatry Res.

[56] Bringmann LF, Elmer T, Epskamp S, Krause RW, Schoch D, Wichers M, Wigman JTW, Snippe E (2019). What do centrality measures measure in psychological networks?. J Abnorm Psychol.

[57] Opsahl T, Agneessens F, Skvoretz J (2010). Node centrality in weighted networks: generalizing degree and shortest paths. Social Networks.

[58] Bringmann LF, Vissers N, Wichers M, Geschwind N, Kuppens P, Peeters F, Borsboom D, Tuerlinckx F (2013). A network approach to psychopathology: new insights into clinical longitudinal data. PLoS One.

[59] Epskamp S, Waldorp LJ, Mõttus R, Borsboom D (2018). The Gaussian graphical model in cross-sectional and time-series data. Multivariate Behav Res.

[60] Fried E, Papanikolaou F, Epskamp S (2021). Mental health and social contact during the COVID-19 pandemic: an ecological momentary assessment study. Clin Psychol Sci.

[61] Reger MA, Stanley IH, Joiner TE (2020). Suicide mortality and coronavirus disease 2019-a perfect storm?. JAMA Psychiatry.

[62] Cacioppo JT, Hawkley LC, Ernst JM, Burleson M, Berntson GG, Nouriani B, Spiegel D (2006). Loneliness within a nomological net: an evolutionary perspective. J Res Person.

[63] Borawski D (2019). Authenticity and rumination mediate the relationship between loneliness and well-being. Curr Psychol.

[64] Pels F, Kleinert J (2016). Loneliness and physical activity: a systematic review. Int Rev Sport Exerc Psychol.

[65] Russell DW, Cutrona CE, McRae C, Gomez M (2012). Is loneliness the same as being alone?. J Psychol.

[66] Torous J, Wykes T (2020). Opportunities From the coronavirus disease 2019 pandemic for transforming psychiatric care with telehealth. JAMA Psychiatry.

[67] Reinhardt I, Gouzoulis-Mayfrank E, Zielasek J (2019). Use of telepsychiatry in emergency and crisis intervention: current evidence. Curr Psychiatry Rep.

[68] Torous J, Jän Myrick K, Rauseo-Ricupero N, Firth J (2020). Digital mental health and COVID-19: using technology today to accelerate the curve on access and quality tomorrow. JMIR Ment Health.

[69] Cohen J, Torous J (2019). The potential of object-relations theory for improving engagement with health apps. JAMA.

[70] Philip P, Dupuy L, Morin CM, de Sevin E, Bioulac S, Taillard J, Serre F, Auriacombe M, Micoulaud-Franchi J (2020). Smartphone-based virtual agents to help individuals with sleep concerns during COVID-19 confinement: feasibility study. J Med Internet Res.

[71] Shaughnessy J, Zechmeister E, Zechmeister J (2000). Research methods in psychology.

[72] Wichers M, Wigman JTW, Bringmann LF, de Jonge P (2017). Mental disorders as networks: some cautionary reflections on a promising approach. Soc Psychiatry Psychiatr Epidemiol.

