# Supplementary material for: The Impact of COVID-19 Lockdown on Daily Activities, Cognitions, and Stress in a Lonely and Distressed Population: Temporal Dynamic Network Analysis
Source: J Med Internet Res. 2022 Mar 17;24(3):e32598. doi: 10.2196/32598 (PMC8972118; doi:10.2196/32598)
Supplement: Multimedia Appendix 1 [file jmir_v24i3e32598_app1.docx]

**Supplement A: Lockdown Stages**

Summary of most important changes in public health measures to counteract the pandemic in Germany between August 2020 and March 2021 (End of measurement, not end of lockdown) (https://www.deutschland.de/en/news/german-federal-government-informs-about-the-corona-crisis)

| **No-Lockdown Stage** | **Lockdown Stage** |
| --- | --- |
| 8 August 2020 – 1 November 2020 | 2 November 2020 - 9 March 2021 |
| 1. Nationwide, a distance of at least 1.5 meters must be maintained, hygiene rules must be observed, and masks must be worn in shops and on public transport. There was no general restriction on public meetings.  2. Institutions and leisure facilities (i.e., theatres, concert halls, cinemas and fitness studios) opened.  3. Sports and recreational activities indoor and outdoor were permitted.  4. Restaurants, bars, pubs, cafés and other catering establishments opened.  All of above-mentioned policies must act in strict compliance with hygiene and infection control regulations  5. Governmental financial aid for those that suffered economic losses during the time of the pandemic. | 1. Only 2 households are allowed to meet, maximal 10 people.  2. Institutions and leisure facilities (i.e., theatres, concert halls, cinemas and fitness studios) had to close.  3. Sports and recreational activities indoor and outdoor were not allowed.  4. All service sectors are closed (e.g., tattoo shops, cosmetic shop), except hairdresser and medically needed treatment, such as ergo- or physiotherapy.  5. Travel restriction abroad and inland. Hotels and pensions are not allowed to receive guests.  6. Schools and kindergartens remained open, as well as youth welfare services.  7. Home office required if possible.  From 16 December 2020 onwards:  1. Only 2 households are allowed to meet, maximal 5 people.  2. Further closing of service sectors (including hairdresser).  3. Closing of most retail, some exception, such as grocer’s shop, pharmacies, post offices, banks and gas station.  4. Closing of schools and kindergarten. |

**Supplement B. Test for stationarity**

To test for non-stationarity, we calculated a two-level AR(1) model, in which each score of the variable included in our model is regressed on the immediately preceding score of that person, resulting in a moment-to-moment inertia score.

The two level AR(1) model can be formulated as following:

Level 1 : $y_{bi}= \mu_{i}+ \phi_{i} (y_{b-1,i}-\mu_{i})+e_{bi}$

Level 2 : $\mu_{i}= \gamma_{00}+ \mu_{0i}$

$\phi_{i} = \gamma_{10}+\mu_{i1}$

Where $\mu_{0}$ represents th mean/trait level of person i, $e_{bi}$ their deviation from this trait level at measurement occasion b that cannot be explained by the autoregression. The lagged Predictor $(y_{b-1,i}-\mu_{i})$ is centered around the person’s trait level and the parameter $\phi_{i}$ represents how much the variable affects itself from one moment to the next moment. Each variable was square root transformed to achieve a normal distribution. The R code for the test for stationarity can be found online at https://osf.io/zskgm/.

Moment-to-moment inertia larger than 1 indicate a non-stationary process ^1^ results are shown in **Table 1**. We see that the average moment-to-moment inertia is between 0.13 and 0.37 for all 7 included variables, with standard errors ranging from 0.002 to 0.18.

| **Group** | **Two-level AR mode** | **Notion** | **Perceived** **restriction** | **Corona**  **Worry** | **Information seeking** | **Stress** | **Loneliness** | **Social activity** | **Physical activity** |
| --- | --- | --- | --- | --- | --- | --- | --- | --- | --- |
| **No-lockdown** | Avg. trait level | $\gamma_{00}$ | 3.80 (0.18) | 3.88  (0.19) | 3.68  (0.17) | 5.11  (0.17) | 3.72  (0.20) | 1.50  (0.03) | 0.19  (0.003) |
|  | Avg. moment-to-moment inertia | $\gamma_{10}$ | 0.26 (0.02) | 0.25  (0.02) | 0.19  (0.02) | 0.28  (0.02) | 0.28  (0.02) | 0.24  (0.02) | 0.16  (0.02) |
| **Lockdown** | Avg. trait level | $\gamma_{00}$ | 4.35  (0.18) | 4.40  (0.18) | 3.68  (0.16) | 4.85  (0.17) | 3.54  (0.21) | 1.66  (0.06) | 0.178  (0.002) |
|  | Avg. moment-to-moment inertia | $\gamma_{10}$ | 0.27  (0.02) | 0.24  (0.02) | 0.13  (0.02) | 0.37  (0.02) | 0.29  (0.02) | 0.26  (0.02) | 0.16  (0.02) |

**Table 1**. Overview of average trait level and averaged moment-to-moment inertia for each of the 7 variable and for each lockdown group. The standard errors for the fixed effects are given between parentheses.

**Supplement C. Permutation Procedure.**

**Centrality indices.** The centrality indices in-strength and out-strength were used in this study. These centrality indices were based on all estimated coefficient in the multilevel autoregressive model (including non-significant one’s). These measures can quantify the importance of each variable within the network.^5^ In-strength reflect the sum of ingoing absolute edge weights (i.e., the sum of predictor’s coefficient for a specific outcome) and out-strength reflect the sum of outgoing absolute edge weights to the specific node (i.e., the sum of the coefficients between specific predictor and all other outcomes). To create a permutation distribution, the group label was randomly assigned to participants, then centrality indices were calculated (i.e., in-strength and out-strength), this was repeated 100,000 times. Statistical significance was determined by counting the occurrence of out-strength/in-strength difference as extreme or more extreme than the differences based on the observed data. This count was divided by the total amount of iteration and doubled to gain a two-sided p-value. We considered difference scores with a (two-sided) p-value below 0.05 as statistically significant.

**Edge differences**. Statistical significance for group differences in network edges was determined by comparing the size of the edge-differences based on the actual data to a permutation distribution. To create a permutation distribution, the group label was randomly assigned to participants, then random coefficients were taken from both groups. This was repeated 100,000 times. Statistical significance was determined by counting the occurrence of coefficient difference as extreme or more extreme than the differences based on the observed data. This count was divided by the total amount of iteration and doubled to gain a two-sided p-value. We considered differences with a (two-sided) p-value below 0.05 as statistically significant.

**Exploratory Analysis: Overall connectivity**. Overall network connectivity was calculated as the mean strength of absolute connection of weight between nodes.^2^ Networks with stronger overall connectivity are thought to be more vulnerable, as nodes are more likely to trigger each other more easily and strongly.^3^ Previous studies have found that stronger overall connectivity signal vulnerability for psychopathology.^2,4^ Group differences in connectivity were calculated by subtracting the connectivity estimates of the no-lockdown stage from the connectivity estimates of the lockdown stage. First, we saved connectivity differences based upon the regression coefficients from a model with the actual data. To create a distribution under a null hypothesis, the group variable (Lockdown, No-lockdown) was randomly assigned to individuals, and subsequently connectivity differences were estimated based on regression coefficients derived from modelling the reshuffled data. This was repeated 100,000 times, statistical significance was determined by counting the occurrence of connectivity difference as extreme or more extreme than the connectivity differences based on the observed data. This count was divided by the total amount of iteration and doubled to gain a two-sided p-value. We considered differences with a (two-sided) p-value below 0.05 as statistically significant.

Permutation tests revealed no statistically significant difference between the two groups in overall network connectivity (i.e., absolute values of all edges; difference = -0.403, *P* = .514; no-lockdown group B= 1.31; lockdown group B= .91). These two groups did also not differ significantly in inter-node connectivity (i.e., cross-regressive edges; difference = .0028, *P* = .865; no-lockdown B = .211; lockdown B= .208) nor intra-node connectivity (i.e., autoregressive effects; difference = .4692, *P* = .515; no-lockdown B= 1.49; lockdown B= 1.03) (see Supplement D).

**Supplement D. Overall connectivity permutation test results between no-lockdown and lockdown group.**

|  | **b.diff.obs** | **b.nolockdown** | **b.lockdown** | **p-perm.def2** |
| --- | --- | --- | --- | --- |
| grp1_vs_grp2_all | 0.4025 | 1.311358 | 0.9088197 | 0.5144 |
| grp1_vs_grp2_diag | 0.0028 | 0.210771 | 0.2080003 | 0.8651 |
| grp1_vs_grp2_off | 0.4692 | 1.494789 | 1.025623 | 0.5148 |

**Supplement E. Permutation results centrality indices in-strength and out-strength between no-lockdown and lockdown group.**

| **Variables** | **Out-Strength** | | | **In-Strength** | | |
| --- | --- | --- | --- | --- | --- | --- |
|  |  | **No-lockdown** | **Lockdown** |  | **No-lockdown** | **Lockdown** |
|  | **Difference** | **B** | **B** | **Difference** | **B** | **B** |
| Perceived restriction | -0.063063 | 0.2542236 | 0.3172868 | 4.748569 | 5.588932 | 0.8403637 |
| Corona worry | 0.041758 | 0.3822933 | 0.3405358 | 2.738025 | 7.007971 | 4.269947 |
| Information seeking | **0.145194*** | 0.3129435 | 0.1677495 | 20.103076 | 23.43126 | 3.32818 |
| Stress | -0.120391 | 0.4190705 | 0.5394615 | -10.880732 | 3.980051 | 14.86078 |
| Loneliness | **-0.197533*** | 0.4109139 | 0.6084465 | 3.245508 | 20.088 | 16.84249 |
| Social activity | -0.757939 | 0.9260614 | 1.684 | -0.254114 | 4.046562 | 4.300676 |
| Physical activity | 20.676352 | 61.55104 | 40.87468 | 0.024048 | 0.1137732 | 0.08972553 |

**Supplement Table E**. Permutation results of centrality indices in-strength and out-strength between no-lockdown and lockdown group. Results that are statistically significant (two-sided p value at the uncorrected α level) are marked in bold and with asterisk.

**Supplement F. Permutation results of dynamic associations between variables for the no-lockdown and lockdown group.**

|  | **No-lockdown** | **Lockdown** | **Difference No-Lockdown vs. Lockdown** |
| --- | --- | --- | --- |
| **Perceived restriction (outcome)** |  |  |  |
| Perceived restriction | 0.168361167 | 0.209280080 | -0.0409 |
| Corona worry | 0.093930687 | 0.058423729 | 0.0355 |
| Information seeking | 0.054766023 | -0.006164548 | **0.0609 *** |
| Stress | 0.057600390 | 0.076000884 | -0.0184 |
| loneliness | 0.000990697 | 0.114984496 | **-0.114*** |
| Social activity | -0.034224490 | -0.199609564 | 0.1654 |
| Physical activity | -5.179058957 | -0.175900440 | -5.0032 |
| **Corona worry**  **(outcome)** |  |  |  |
| Perceived restriction | 0.04961000 | 0.03815016 | 0.0115 |
| Corona worry | 0.19130966 | 0.17582498 | **0.0155*** |
| Information seeking | 0.06893197 | 0.02120137 | **0.0477*** |
| Stress | 0.02685971 | 0.05706330 | -0.0302 |
| loneliness | 0.02744843 | 0.10418599 | **-0.0767*** |
| Social activity | -0.01084773 | -0.17633630 | 0.1655 |
| Physical activity | -6.63296364 | -3.69718455 | -2.9358 |
| **Information seeking** |  |  |  |
| Perceived restriction | -0.009187084 | -0.03182693 | 0.0226 |
| Corona worry | 0.068932020 | 0.06828975 | 6e-04 |
| Information seeking | 0.172070557 | 0.09671964 | **0.0754*** |
| Stress | 0.016648518 | 0.01513835 | 0.0015 |
| loneliness | -0.004302592 | 0.04524142 | -0.0495 |
| Social activity | -0.089219118 | -0.19994264 | 0.1107 |
| Physical activity | -23.070896004 | -2.87102164 | -20.1999 |
| **Stress** |  |  |  |
| Perceived restriction | 0.009905426 | 0.029978702 | -0.0201 |
| Corona worry | 0.014639647 | -0.004817997 | 0.0195 |
| Information seeking | 0.013378323 | 0.026506587 | -0.0131 |
| Stress | 0.281292472 | 0.349809306 | -0.0685 |
| loneliness | 0.082897759 | 0.076983545 | 0.0059 |
| Social activity | -0.236783136 | -0.508308587 | 0.2715 |
| Physical activity | 3.341154199 | -13.864377864 | 17.2055 |
| **Loneliness** |  |  |  |
| Perceived restriction | 0.01281949 | 0.005914085 | 0.0069 |
| Corona worry | -0.01293064 | 0.031500069 | **-0.0444*** |
| Information seeking | -0.00250844 | -0.016125181 | 0.0136 |
| Stress | 0.03531339 | 0.038755178 | -0.0034 |
| loneliness | 0.29161681 | 0.266552851 | 0.0251 |
| Social activity | -0.29704402 | -0.331087923 | 0.034 |
| Physical activity | -19.43576336 | -16.152552853 | -3.2832 |
| **Social activity** |  |  |  |
| Perceived restriction | 0.0043388190 | -0.0021206610 | **0.0065*** |
| Corona worry | -0.0005405175 | 0.0016629806 | -0.0022 |
| Information seeking | -0.0012475821 | -0.0010179940 | -2e-04 |
| Stress | -0.0013483639 | -0.0026195790 | 0.0013 |
| loneliness | -0.0036485525 | -0.0004227891 | -0.0032 |
| Social activity | 0.2574926656 | 0.2685007186 | -0.011 |
| Physical activity | 3.7779456112 | 4.0243313946 | -0.2464 |
| **Physical activity** |  |  |  |
| Perceived restriction | 0.000001567147 | 0.00001617310 | -1.460595e-05 |
| Corona worry | 0.000010136284 | 0.00001625251 | -6.116226e-06 |
| Information seeking | 0.000040654396 | -0.00001414608 | 5.480048e-05 |
| Stress | 0.000007707282 | 0.00007489472 | -6.718744e-05 |
| loneliness | -0.000009045968 | -0.00007541477 | 6.63688e-05 |
| Social activity | 0.000450261913 | 0.00021419024 | 0.0002360717 |
| Physical activity | 0.113253876221 | 0.08931445475 | 0.0239 |

**Supplement Table F**. Associations between variables for the no-lockdown and lockdown group, and the differences in associations between groups. Results that are statistically significant (permutation testing using two-sided p value at the uncorrected α level) are marked with bold font and asterisks.

**Supplement G. Testing for the effect of gender.**

Because we had more female participants in our lockdown group compared to the no-lockdown group, we are tested the effect of gender on each measured variable. For each participant, we calculated an average score for every variable included in our network analyses. Gender consisted of three levels: male, female and diverse.

*Stress*. A one-way ANOVA revealed that there was no significant difference of stress scores among different genders (*F*(2, 255) = 0.27, *P* = 0.764).

*COVID-19 related worry*. A one-way ANOVA revealed that there was no significant difference of levels of COVID-19 related worry among different genders (*F*(2, 255) = 0.727, *P* = 0.484).

*Perceived restriction*. A one-way ANOVA revealed that there was no significant difference of levels of perceived restriction among different genders (*F*(2, 255) = 0.961, *P* = 0.384).

*Information seeking*. A one-way ANOVA revealed that there was no significant difference of levels of information seeking among different genders (*F*(2, 255) = 0.294, *P* = 0.745).

*Loneliness*. A one-way ANOVA revealed that there was no significant difference of loneliness scores among different genders (*F(*2, 255) = 2.757, *P* = 0.065).

*Physical activity*. A one-way ANOVA revealed that there was no significant difference of levels of perceived restriction among different genders (*F*(2, 255) = 0.297, *P* = 0.743).

*Social activity*. A one-way ANOVA revealed that there was no significant difference of duration of social activity among different genders (*F*(2, 255) = 11.62, *P* = < 0.001). Tukey’s HSD Test for multiple comparisons found that females spent more time on social activity than males (*P* < 0.001, 95% C.I. of difference score = [0.3095, 0.9228], Male: *M* = 2.41, *SD* = 0.886, Female: *M* = 3.02, *SD* = 0.886, Diverse: M = 3.33, SD = 1.17).

**References**

1. de Haan-Rietdijk S, Kuppens P, Hamaker EL. What's in a day? A guide to decomposing the variance in intensive longitudinal data. *Frontiers in Psychology.* 2016;7:891.

2. Bringmann LF, Pe ML, Vissers N, et al. Assessing temporal emotion dynamics using networks. *Assessment.* 2016;23(4):425-435.

3. Cramer AO, Van Borkulo CD, Giltay EJ, et al. Major depression as a complex dynamic system. *PLOS ONE.* 2016;11(12):e0167490.

4. Pe ML, Kircanski K, Thompson RJ, et al. Emotion-network density in major depressive disorder. *Clinical Psychological Science.* 2015;3(2):292-300.

5. Opsahl T, Agneessens F, Skvoretz J. Node centrality in weighted networks: Generalizing degree and shortest paths. *Social Networks.* 2010;32(3):245-251.
